# Dipotassium diaqua­bis(methyl­enedi­phospho­nato-κ^2^ *O*,*O*′)cobaltate(II)

**DOI:** 10.1107/S160053680905106X

**Published:** 2010-01-16

**Authors:** H.G. Visser, J.A. Venter, K.A. Van der Merwe

**Affiliations:** a Department of Chemistry, University of the Free State, PO Box 339, Bloemfontein 9300, South Africa

## Abstract

In the title complex, K_2_[Co(CH_4_O_6_P_2_)_2_(H_2_O)_2_], the asymmetric unit contains two K^+^ cations and two half-anions in which the Co atoms lie on inversion centers. The Co^II^ ions assume an octa­hedral CoO_6_ coordination geometry. In the crystal, a three-dimensional network is formed through O—H⋯O hydrogen-bond inter­actions as well as inter­molecular inter­actions between the K^+^ cations and neighbouring O atoms.

## Related literature

For related structures, see: DeLaMatter *et al.* (1973 ▶); Jurisson *et al.* (1983 ▶); Barthelet *et al.* (2002 ▶); Stahl *et al.* (2006 ▶); Van der Merwe *et al.* (2009 ▶).
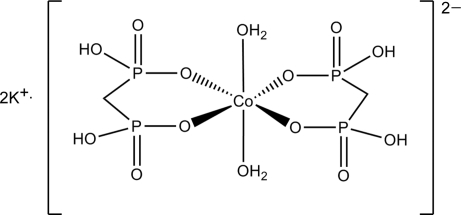


## Experimental

### 

#### Crystal data

K_2_[Co(CH_4_O_6_P_2_)_2_(H_2_O)_2_]
*M* *_r_* = 521.13
Triclinic, 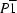

*a* = 6.4523 (3) Å
*b* = 8.7056 (3) Å
*c* = 13.1930 (5) Å
α = 91.334 (2)°
β = 93.304 (2)°
γ = 93.333 (2)°
*V* = 738.32 (5) Å^3^
*Z* = 2
Mo *K*α radiation
μ = 2.23 mm^−1^
*T* = 100 K
0.28 × 0.17 × 0.17 mm

#### Data collection

Bruker X8 APEXII 4K Kappa CCD diffractometer
Absorption correction: multi-scan *SADABS* (Bruker, 2004 ▶) *T*_min_ = 0.635, *T*_max_ = 0.690
13474 measured reflections
3645 independent reflections
3194 reflections with *I* > 2σ(*I*)
*R*_int_ = 0.036

#### Refinement

*R*[*F* ^2^ > 2σ(*F* ^2^)] = 0.040
*wR*(*F* ^2^) = 0.1096
*S* = 1.23
3645 reflections
235parameters
14 restraints
H atoms treated by a mixture of independent and constrained refinement
Δρ_max_ = 1.04 e Å^−3^
Δρ_min_ = −0.61 e Å^−3^

### 

Data collection: *APEX2* (Bruker, 2005 ▶); cell refinement: *SAINT-Plus* (Bruker, 2004 ▶); data reduction: *SAINT-Plus* and *XPREP* (Bruker, 2004 ▶); program(s) used to solve structure: *SIR97* (Altomare *et al.*, 1999 ▶); program(s) used to refine structure: *SHELXL97* (Sheldrick, 2008 ▶); molecular graphics: *DIAMOND* (Brandenburg & Putz, 2005 ▶); software used to prepare material for publication: *WinGX* (Farrugia, 1999 ▶).

## Supplementary Material

Crystal structure: contains datablocks global, I. DOI: 10.1107/S160053680905106X/ds2013sup1.cif

Structure factors: contains datablocks I. DOI: 10.1107/S160053680905106X/ds2013Isup2.hkl

Additional supplementary materials:  crystallographic information; 3D view; checkCIF report

## Figures and Tables

**Table 1:** Selected bond lengths (Å)

O1—Co1	2.052 (3)
O2—Co1	2.132 (2)
O3—Co1	2.127 (3)
O8—Co2	2.081 (3)
O9—Co2	2.117 (2)
O10—Co2	2.064 (3)

**Table 2:** Hydrogen-bond geometry (Å, °)

*D*—H⋯*A*	*D*—H	H⋯*A*	*D*⋯*A*	*D*—H⋯*A*
O3—H3*A*⋯O14^i^	0.85 (2)	1.83 (2)	2.680 (4)	175 (4)
O3—H3*B*⋯O6^ii^	0.86 (2)	1.89 (2)	2.737 (4)	172 (4)
O4—H4⋯O9^iii^	0.84 (2)	1.81 (2)	2.632 (4)	166 (5)
O7—H7⋯O6^iv^	0.85 (2)	1.72 (2)	2.570 (4)	177 (5)
O8—H8*A*⋯O5^v^	0.85 (2)	1.84 (2)	2.678 (4)	170 (4)
O8—H8*B*⋯O11^vi^	0.85 (2)	1.84 (2)	2.687 (4)	176 (5)
O12—H12⋯O11^vii^	0.85 (1)	1.72 (1)	2.561 (4)	175 (5)
O13—H13⋯O2^i^	0.84 (2)	1.78 (2)	2.616 (4)	174 (5)

Symmetry codes: (i) 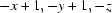; (ii) 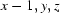; (iii) 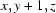; (iv) 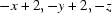; (v) 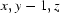; (vi) 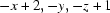; (vii) 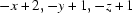.

**Fig. 1.**
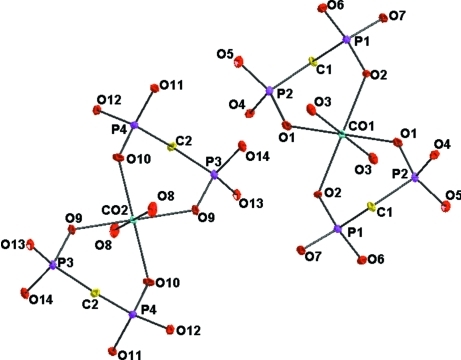
View of (I) (50% probability displacement ellipsoids). The potassium cations and hydrogen atoms have been omitted for clarity.

## supplementary crystallographic information

### Comment

We reported a similar structure recently with the only differences being the cation and the +2 oxidation state of the cobalt ion (Van der Merwe et al.<i/>, 2009).

The Co^III^ ion in the title complex, K[Co(C_2_H_4_O_6_P)_2_(H_2_O)_2_], is in a slightly distorted octahedral environment with O–Co–O bond angles varying from 83.75 (10) to 96.25 (10)°.  All the bonding distance and angles fall within the normal range observed for complexes of this nature.  The P-O distances are significantly different for P═O and P-OH type bonds and vary from 1.501 (3) to 1.580 (3) Å.  This could possibly be an indication that the assignment of  positional disorders for the respective Co^II^ complex previously was correct since these difference were not so prominent in the previous structure.

A three-dimensional network is provided by numerous hydrogen bonds and other weak interactions between the potassium ions and the oxygen atoms of the anionic species.

### Experimental

CoCl_2_.6H_2_O (0,1696 g, 71 mmol)  was dissolved in water (7 cm^3^) and heated to 70°C.  Potassium bicarbonate was added to raise the pH to 5,5 after which methylene disphosphonate (0,25 g, 142 mmol), dissolved in water (5 cm^3^) was added dropwise.  The final pH of the solution was adjusted to 1.50 to obtain the Co^II^ salt as described previously (Van der Merwe et al.<i/> 2009).  Crystals of the Co^III^ salt, suitable for X-ray diffraction, was obtained from redissolving and adding H_2_O_2_ to the solution.

### Refinement

The aliphatic H atoms were placed in geometrically idealized positions and constrained to ride on their parent atoms with *U*_iso_(H) = 1.2*U*_eq_(C). The hydroxyl and aqua ions were located from the difference Fourier map. The highest residual electron density was located 1.40 Å from O1 and the deepest hole was 0.64 Å from Co1.

### Crystal data

**Table:**

K_2_[Co(CH_4_O_6_P_2_)_2_(H_2_O)_2_]	*Z* = 2
*M**_r_* = 521.13	*F*(000) = 522
Triclinic, *P*1	*D*_x_ = 2.344 Mg m^−^^3^
Hall symbol: -P 1	Mo *K*α radiation, λ = 0.71073 Å
*a* = 6.4523 (3) Å	Cell parameters from 5231 reflections
*b* = 8.7056 (3) Å	θ = 2.1–28.3°
*c* = 13.1930 (5) Å	µ = 2.23 mm^−^^1^
α = 91.334 (2)°	*T* = 100 K
β = 93.304 (2)°	Cuboid, pink
γ = 93.333 (2)°	0.28 × 0.17 × 0.17 mm
*V* = 738.32 (5) Å^3^	

### Data collection

**Table:**

Bruker X8 APEXII 4K Kappa CCD diffractometer	3645 independent reflections
Radiation source: sealed tube	3194 reflections with *I* > 2σ(*I*)
graphite	*R*_int_ = 0.036
phi and ω scans	θ_max_ = 28.4°, θ_min_ = 3.1°
Absorption correction: multi-scan *SADABS* (Bruker, 2004)	*h* = −8→8
*T*_min_ = 0.635, *T*_max_ = 0.690	*k* = −11→9
13474 measured reflections	*l* = −16→17

### Refinement

**Table:**

Refinement on *F*^2^	14 restraints
Least-squares matrix: full	H atoms treated by a mixture of independent and constrained refinement
*R*[*F*^2^ > 2σ(*F*^2^)] = 0.040	*w* = 1/[σ^2^(*F*_o_^2^) + (0.0164*P*)^2^ + 3.9058*P*] where *P* = (*F*_o_^2^ + 2*F*_c_^2^)/3
*wR*(*F*^2^) = 0.096	(Δ/σ)_max_ < 0.001
*S* = 1.18	Δρ_max_ = 0.95 e Å^−^^3^
3645 reflections	Δρ_min_ = −0.55 e Å^−^^3^
235 parameters	

### Special details

**Table:**

Geometry. All e.s.d.'s (except the e.s.d. in the dihedral angle between two l.s. planes) are estimated using the full covariance matrix. The cell e.s.d.'s are taken into account individually in the estimation of e.s.d.'s in distances, angles and torsion angles; correlations between e.s.d.'s in cell parameters are only used when they are defined by crystal symmetry. An approximate (isotropic) treatment of cell e.s.d.'s is used for estimating e.s.d.'s involving l.s. planes.

### Fractional atomic coordinates and isotropic or equivalent isotropic displacement parameters (Å^2^)

**Table:**

	*x*	*y*	*z*	*U*_iso_*/*U*_eq_	
C2	0.6164 (6)	0.3660 (4)	0.4121 (3)	0.0083 (7)	
H2A	0.6647	0.4636	0.3852	0.01*	
H2B	0.4955	0.3844	0.4501	0.01*	
O2	0.7149 (4)	0.6643 (3)	−0.06002 (19)	0.0084 (5)	
O1	0.4857 (4)	0.6095 (3)	0.1386 (2)	0.0100 (5)	
O4	0.3898 (4)	0.8338 (3)	0.2356 (2)	0.0093 (5)	
O5	0.7656 (4)	0.7614 (3)	0.2543 (2)	0.0116 (5)	
O6	1.0177 (4)	0.7998 (3)	0.0450 (2)	0.0088 (5)	
O8	0.6907 (4)	−0.1647 (3)	0.4464 (2)	0.0137 (6)	
O9	0.4598 (4)	0.0840 (3)	0.35124 (19)	0.0087 (5)	
O10	0.7627 (4)	0.1422 (3)	0.5352 (2)	0.0109 (5)	
O11	1.0208 (4)	0.3123 (3)	0.4451 (2)	0.0091 (5)	
O12	0.8299 (4)	0.4173 (3)	0.59244 (19)	0.0097 (5)	
O13	0.3411 (4)	0.3121 (3)	0.2569 (2)	0.0094 (5)	
O14	0.7069 (4)	0.2216 (3)	0.2347 (2)	0.0105 (5)	
P1	0.80660 (14)	0.81239 (10)	−0.00988 (7)	0.00684 (18)	
P2	0.57617 (14)	0.76121 (11)	0.18262 (7)	0.00712 (18)	
P3	0.53703 (14)	0.23561 (10)	0.30689 (7)	0.00681 (18)	
P4	0.81776 (14)	0.30133 (11)	0.49860 (7)	0.00724 (18)	
K1	0.08042 (13)	0.57684 (9)	0.19914 (6)	0.01125 (17)	
K2	0.02621 (13)	0.04058 (10)	0.31295 (7)	0.01350 (18)	
Co1	0.5	0.5	0	0.00676 (15)	
Co2	0.5	0	0.5	0.00634 (15)	
O7	0.8190 (4)	0.9319 (3)	−0.0975 (2)	0.0092 (5)	
O3	0.2452 (4)	0.6119 (3)	−0.0684 (2)	0.0112 (5)	
C1	0.6299 (6)	0.8839 (4)	0.0776 (3)	0.0079 (7)	
H1A	0.4994	0.9006	0.0404	0.01*	
H1B	0.6864	0.9831	0.1048	0.01*	
H8B	0.778 (6)	−0.215 (5)	0.482 (3)	0.02*	
H8A	0.699 (7)	−0.185 (5)	0.3838 (14)	0.02*	
H7	0.877 (7)	1.020 (3)	−0.081 (4)	0.02*	
H4	0.433 (8)	0.911 (4)	0.271 (3)	0.02*	
H13	0.332 (8)	0.318 (6)	0.1935 (15)	0.02*	
H3B	0.175 (7)	0.665 (5)	−0.029 (3)	0.02*	
H3A	0.266 (8)	0.661 (5)	−0.122 (2)	0.02*	
H12	0.872 (7)	0.5089 (18)	0.581 (2)	0.02*	

### Atomic displacement parameters (Å^2^)

**Table:**

	*U*^11^	*U*^22^	*U*^33^	*U*^12^	*U*^13^	*U*^23^
C2	0.0098 (17)	0.0062 (16)	0.0087 (16)	−0.0003 (13)	−0.0010 (13)	0.0003 (13)
O2	0.0098 (12)	0.0077 (12)	0.0073 (12)	−0.0033 (10)	0.0005 (10)	0.0002 (9)
O1	0.0146 (13)	0.0065 (12)	0.0089 (12)	−0.0016 (10)	0.0035 (10)	0.0000 (10)
O4	0.0087 (12)	0.0097 (13)	0.0092 (12)	0.0009 (10)	−0.0002 (10)	−0.0023 (10)
O5	0.0108 (13)	0.0146 (13)	0.0095 (13)	0.0031 (10)	−0.0004 (10)	−0.0010 (10)
O6	0.0082 (12)	0.0078 (12)	0.0102 (12)	−0.0004 (9)	−0.0006 (10)	−0.0002 (10)
O8	0.0150 (14)	0.0183 (14)	0.0087 (13)	0.0097 (11)	−0.0005 (11)	−0.0010 (11)
O9	0.0138 (13)	0.0062 (12)	0.0060 (12)	−0.0009 (10)	0.0005 (10)	0.0016 (9)
O10	0.0088 (12)	0.0092 (12)	0.0146 (13)	−0.0013 (10)	−0.0011 (10)	0.0065 (10)
O11	0.0084 (12)	0.0096 (12)	0.0092 (12)	−0.0005 (10)	0.0020 (10)	0.0003 (10)
O12	0.0139 (13)	0.0077 (12)	0.0072 (12)	−0.0016 (10)	0.0002 (10)	−0.0008 (10)
O13	0.0109 (13)	0.0116 (13)	0.0060 (12)	0.0023 (10)	−0.0004 (10)	0.0006 (10)
O14	0.0118 (13)	0.0127 (13)	0.0075 (12)	0.0023 (10)	0.0023 (10)	0.0021 (10)
P1	0.0077 (4)	0.0062 (4)	0.0067 (4)	0.0000 (3)	0.0006 (3)	0.0005 (3)
P2	0.0080 (4)	0.0076 (4)	0.0059 (4)	0.0014 (3)	0.0010 (3)	−0.0004 (3)
P3	0.0086 (4)	0.0063 (4)	0.0057 (4)	0.0013 (3)	0.0005 (3)	0.0006 (3)
P4	0.0083 (4)	0.0063 (4)	0.0069 (4)	−0.0006 (3)	−0.0010 (3)	0.0008 (3)
K1	0.0112 (4)	0.0122 (4)	0.0107 (4)	0.0026 (3)	0.0011 (3)	0.0015 (3)
K2	0.0109 (4)	0.0126 (4)	0.0167 (4)	0.0017 (3)	−0.0022 (3)	−0.0013 (3)
Co1	0.0083 (3)	0.0060 (3)	0.0061 (3)	0.0004 (2)	0.0010 (2)	−0.0001 (2)
Co2	0.0072 (3)	0.0061 (3)	0.0058 (3)	0.0001 (2)	0.0008 (2)	0.0007 (2)
O7	0.0137 (13)	0.0055 (12)	0.0081 (12)	−0.0021 (10)	0.0003 (10)	0.0016 (10)
O3	0.0123 (13)	0.0130 (13)	0.0091 (13)	0.0030 (10)	0.0036 (10)	0.0028 (10)
C1	0.0104 (17)	0.0061 (16)	0.0077 (16)	0.0015 (13)	0.0016 (13)	0.0017 (13)

### Geometric parameters (Å, °)

**Table:**

C2—P4	1.803 (4)	O13—K1	3.018 (3)
C2—P3	1.804 (4)	O13—K2	3.156 (3)
C2—H2A	0.97	O13—H13	0.837 (19)
C2—H2B	0.97	O14—P3	1.502 (3)
O2—P1	1.509 (3)	O14—K2^ii^	2.829 (3)
O1—Co1	2.052 (3)	P1—O7	1.575 (3)
O2—Co1	2.132 (2)	P1—C1	1.795 (4)
O3—Co1	2.127 (3)	P2—C1	1.806 (4)
O1—P2	1.503 (3)	K1—O12^v^	2.776 (3)
O1—K1	2.779 (3)	K1—O5^vi^	2.780 (3)
O4—P2	1.581 (3)	K1—O6^vi^	2.871 (3)
O4—K1	2.922 (3)	K1—O3^vii^	3.023 (3)
O4—K2^i^	3.235 (3)	K1—O2^viii^	3.153 (3)
O4—H4	0.84 (2)	K2—O14^vi^	2.829 (3)
O5—P2	1.501 (3)	K2—O11^vi^	2.909 (3)
O5—K1^ii^	2.780 (3)	K2—O10^iv^	2.913 (3)
O5—K2^iii^	2.936 (3)	K2—O5^ix^	2.936 (3)
O6—P1	1.516 (3)	K2—O7^viii^	3.076 (3)
O6—K1^ii^	2.871 (3)	K2—O4^x^	3.235 (3)
O8—Co2	2.081 (3)	K2—O8^vi^	3.334 (3)
O8—K2^ii^	3.334 (3)	Co1—O1^viii^	2.052 (3)
O8—H8B	0.854 (19)	Co1—O3	2.126 (3)
O8—H8A	0.845 (19)	Co1—O3^viii^	2.126 (3)
O9—P3	1.525 (3)	Co1—O2^viii^	2.132 (2)
O9—Co2	2.117 (2)	Co2—O10^iv^	2.064 (3)
O9—K2	2.816 (3)	Co2—O8^iv^	2.081 (3)
O10—P4	1.508 (3)	Co2—O9^iv^	2.117 (2)
O10—Co2	2.064 (3)	O7—K2^viii^	3.076 (3)
O10—K2^iv^	2.913 (3)	O7—H7	0.849 (19)
O11—P4	1.523 (3)	O3—K1^vii^	3.023 (3)
O11—K2^ii^	2.909 (3)	O3—H3B	0.855 (19)
O12—P4	1.575 (3)	O3—H3A	0.851 (19)
O12—K1^v^	2.776 (3)	C1—H1A	0.97
O12—H12	0.848 (12)	C1—H1B	0.97
O13—P3	1.581 (3)		
			
P4—C2—P3	115.32 (19)	P2^vi^—K1—H13	153.4 (10)
P4—C2—H2A	108.4	Co1—K1—H13	54.0 (7)
P3—C2—H2A	108.4	K2^i^—K1—H13	146.4 (9)
P4—C2—H2B	108.4	O9—K2—O14^vi^	135.52 (8)
P3—C2—H2B	108.4	O9—K2—O11^vi^	83.20 (8)
H2A—C2—H2B	107.5	O9—K2—O10^iv^	60.10 (7)
P1—O2—Co1	127.62 (15)	O9—K2—O5^ix^	130.52 (8)
P2—O1—Co1	133.27 (16)	O9—K2—O7^viii^	77.74 (7)
P2—O1—K1	106.53 (13)	O5^ix^—K2—O7^viii^	91.81 (8)
Co1—O1—K1	108.92 (11)	O9—K2—O13	49.14 (7)
P2—O4—K1	98.19 (12)	O14^vi^—K2—O13	86.68 (8)
P2—O4—H4	110 (4)	O11^vi^—K2—O13	66.26 (7)
K1—O4—H4	148 (4)	O10^iv^—K2—O13	107.66 (7)
K2^i^—O4—H4	66 (4)	O5^ix^—K2—O13	150.33 (8)
Co2—O8—H8B	127 (3)	O7^viii^—K2—O13	58.52 (7)
K2^ii^—O8—H8B	99 (3)	O9—K2—O4^x^	50.98 (7)
Co2—O8—H8A	123 (3)	O13—K2—O4^x^	82.11 (7)
K2^ii^—O8—H8A	60 (4)	O9—K2—O8^vi^	127.53 (8)
H8B—O8—H8A	110 (3)	O13—K2—O8^vi^	159.31 (8)
P3—O9—Co2	130.58 (16)	O9—K2—P3	23.35 (5)
P3—O9—K2	109.60 (13)	O14^vi^—K2—P3	112.35 (6)
Co2—O9—K2	101.82 (10)	O11^vi^—K2—P3	73.94 (6)
P4—O10—Co2	129.21 (16)	O10^iv^—K2—P3	82.69 (6)
P4—O12—H12	115.7 (19)	O5^ix^—K2—P3	145.87 (6)
K1^v^—O12—H12	98 (2)	O7^viii^—K2—P3	66.54 (5)
P3—O13—K1	155.08 (15)	O13—K2—P3	25.79 (5)
P3—O13—K2	93.90 (12)	O4^x^—K2—P3	64.15 (5)
K1—O13—K2	106.16 (8)	O8^vi^—K2—P3	147.32 (6)
P3—O13—H13	118 (4)	O9—K2—P4^vi^	103.51 (6)
K1—O13—H13	71 (4)	O13—K2—P4^vi^	88.53 (5)
K2—O13—H13	107 (4)	P3—K2—P4^vi^	97.09 (3)
O2—P1—O6	114.49 (15)	O9—K2—P3^vi^	143.95 (6)
O2—P1—O7	105.55 (15)	P3—K2—P3^vi^	124.45 (3)
O6—P1—O7	111.05 (15)	O1^viii^—Co1—O1	180.00 (7)
O2—P1—C1	109.75 (16)	O1^viii^—Co1—O3	85.70 (11)
O6—P1—C1	109.14 (16)	O1—Co1—O3	94.30 (11)
O7—P1—C1	106.53 (15)	O1—Co1—O3^viii^	85.70 (11)
O5—P2—O1	118.26 (16)	O3—Co1—O3^viii^	180
O5—P2—O4	111.00 (15)	O1^viii^—Co1—O2	83.75 (10)
O1—P2—O4	104.54 (15)	O1—Co1—O2	96.25 (10)
O5—P2—C1	109.50 (17)	O3—Co1—O2	90.87 (10)
O1—P2—C1	107.35 (16)	O3^viii^—Co1—O2	89.13 (10)
O4—P2—C1	105.35 (15)	O1—Co1—O2^viii^	83.75 (10)
O5—P2—K1	130.08 (11)	O3—Co1—O2^viii^	89.13 (10)
O1—P2—K1	49.28 (11)	O2—Co1—O2^viii^	180
O4—P2—K1	55.38 (10)	O1^viii^—Co1—K1	138.32 (8)
C1—P2—K1	120.38 (13)	O1—Co1—K1	41.68 (8)
O14—P3—O9	114.63 (15)	O3—Co1—K1	69.01 (7)
O14—P3—O13	112.37 (15)	O3^viii^—Co1—K1	110.99 (7)
O9—P3—O13	107.36 (15)	O2—Co1—K1	127.38 (7)
O14—P3—C2	111.63 (17)	O2^viii^—Co1—K1	52.62 (7)
O9—P3—C2	107.33 (16)	O1—Co1—K1^viii^	138.32 (8)
O13—P3—C2	102.70 (15)	O3—Co1—K1^viii^	110.99 (7)
O14—P3—K2	131.70 (12)	O2—Co1—K1^viii^	52.62 (7)
O9—P3—K2	47.05 (10)	K1—Co1—K1^viii^	180
O13—P3—K2	60.31 (10)	O10^iv^—Co2—O10	180
C2—P3—K2	116.55 (12)	O10^iv^—Co2—O8	91.42 (11)
O10—P4—O11	113.25 (15)	O10—Co2—O8	88.58 (11)
O10—P4—O12	108.39 (15)	O10—Co2—O8^iv^	91.42 (11)
O11—P4—O12	110.21 (15)	O8—Co2—O8^iv^	180.00 (14)
O10—P4—C2	111.43 (16)	O10^iv^—Co2—O9	86.68 (10)
O11—P4—C2	107.68 (16)	O10—Co2—O9	93.32 (10)
O12—P4—C2	105.61 (16)	O8—Co2—O9	89.71 (10)
O1—K1—O4	50.58 (7)	O8^iv^—Co2—O9	90.29 (10)
O5^vi^—K1—O4	90.83 (8)	O10—Co2—O9^iv^	86.68 (10)
O6^vi^—K1—O4	71.15 (8)	O8—Co2—O9^iv^	90.29 (10)
O12^v^—K1—O13	69.91 (8)	O9—Co2—O9^iv^	180.0000 (10)
O1—K1—O13	66.27 (8)	O10^iv^—Co2—K2	48.07 (7)
O5^vi^—K1—O13	146.12 (8)	O10—Co2—K2	131.93 (7)
O6^vi^—K1—O13	142.50 (8)	O8—Co2—K2	110.65 (8)
O4—K1—O13	99.99 (8)	O8^iv^—Co2—K2	69.35 (8)
O1—K1—P2	24.20 (6)	O9—Co2—K2	45.66 (7)
O5^vi^—K1—P2	116.53 (7)	O9^iv^—Co2—K2	134.34 (7)
O6^vi^—K1—P2	76.27 (6)	O10—Co2—K2^iv^	48.07 (7)
O4—K1—P2	26.43 (5)	O8—Co2—K2^iv^	69.35 (8)
O13—K1—P2	81.17 (6)	O9—Co2—K2^iv^	134.34 (7)
O1—K1—Co1	29.41 (6)	K2—Co2—K2^iv^	180.00 (2)
O5^vi^—K1—Co1	146.14 (6)	P1—O7—K2^viii^	139.92 (14)
O6^vi^—K1—Co1	74.83 (5)	P1—O7—H7	116 (3)
O4—K1—Co1	76.27 (5)	K2^viii^—O7—H7	90 (3)
O13—K1—Co1	67.68 (5)	Co1—O3—K1^vii^	118.88 (11)
O3^vii^—K1—Co1	90.27 (5)	Co1—O3—H3B	117 (3)
O2^viii^—K1—Co1	32.50 (5)	K1^vii^—O3—H3B	104 (3)
P2—K1—Co1	51.534 (19)	Co1—O3—H3A	118 (3)
O1—K1—H13	59.5 (10)	K1^vii^—O3—H3A	85 (3)
O5^vi^—K1—H13	159.3 (8)	H3B—O3—H3A	110 (3)
O6^vi^—K1—H13	127.9 (5)	P1—C1—P2	115.47 (19)
O4—K1—H13	102.4 (10)	P1—C1—H1A	108.4
O13—K1—H13	16.1 (4)	P2—C1—H1A	108.4
O3^vii^—K1—H13	87.6 (9)	P1—C1—H1B	108.4
O2^viii^—K1—H13	34.1 (4)	P2—C1—H1B	108.4
P2—K1—H13	79.2 (10)	H1A—C1—H1B	107.5

Symmetry codes: (i) *x*, *y*+1, *z*; (ii) *x*+1, *y*, *z*; (iii) *x*+1, *y*+1, *z*; (iv) −*x*+1, −*y*, −*z*+1; (v) −*x*+1, −*y*+1, −*z*+1; (vi) *x*−1, *y*, *z*; (vii) −*x*, −*y*+1, −*z*; (viii) −*x*+1, −*y*+1, −*z*; (ix) *x*−1, *y*−1, *z*; (x) *x*, *y*−1, *z*.

### Hydrogen-bond geometry (Å, °)

**Table:**

*D*—H···*A*	*D*—H	H···*A*	*D*···*A*	*D*—H···*A*
O3—H3A···O14^viii^	0.85 (2)	1.83 (2)	2.680 (4)	175 (4)
O3—H3B···O6^vi^	0.86 (2)	1.89 (2)	2.737 (4)	172 (4)
O4—H4···O9^i^	0.84 (2)	1.81 (2)	2.632 (4)	166 (5)
O7—H7···O6^xi^	0.85 (2)	1.72 (2)	2.570 (4)	177 (5)
O8—H8A···O5^x^	0.85 (2)	1.84 (2)	2.678 (4)	170 (4)
O8—H8B···O11^xii^	0.85 (2)	1.84 (2)	2.687 (4)	176 (5)
O12—H12···O11^xiii^	0.85 (1)	1.72 (1)	2.561 (4)	175 (5)
O13—H13···O2^viii^	0.84 (2)	1.78 (2)	2.616 (4)	174 (5)

Symmetry codes: (viii) −*x*+1, −*y*+1, −*z*; (vi) *x*−1, *y*, *z*; (i) *x*, *y*+1, *z*; (xi) −*x*+2, −*y*+2, −*z*; (x) *x*, *y*−1, *z*; (xii) −*x*+2, −*y*, −*z*+1; (xiii) −*x*+2, −*y*+1, −*z*+1.
